# How to Improve the Public Trust of the Intelligent Aging Community: An Empirical Study Based on the ACSI Model

**DOI:** 10.3390/ijerph18041971

**Published:** 2021-02-18

**Authors:** Tuochen Li, Siran Wang

**Affiliations:** School of Economics and Management, Harbin Engineering University, Harbin 150001, China; lituochen0919@163.com

**Keywords:** intelligent aging community, public trust, formation mechanism, ACSI model

## Abstract

In order to enhance social trust in intelligent aging services, the formation mechanism of public trust in the intelligent aging community was studied. Based on the classic American Customer Satisfaction Index (ACSI) model, this paper establishes the public trust formation model of the intelligent aging community by proposing relevant assumptions. Using 306 questionnaires from China’s intelligent aging care model community as the original data, the model is empirically tested through structural equation modeling. The empirical results show that: firstly, the public satisfaction with the intelligent aging community directly determines the formation of public trust, and the key to improving public trust in the intelligent aging community is to improve customer satisfaction. Secondly, perceived quality, perceived ease of use, perceived risk, and perceived cost economical directly affect public satisfaction and indirectly affect the formation process of public trust in the intelligent aging community. Public satisfaction serves as a complete intermediary in this process.

## 1. Introduction

The United Nations considers a society in which more than 7% of the population are aged 65 and above, or more than 10% of the population are aged 60 and above as an aging society. By the end of 2019, China’s population aged 65 and above had reached 175 million, accounting for 12.5% of the total population [1]. Population aging has become a serious social problem in China. With the advent of the aging society and the progress of Internet technology, the ordinary traditional aging service model has been unable to meet the diversified needs of the elderly. With the rapid development of intelligent equipment, digital communication, and other technologies, a new aging service model called “intelligent aging care” has emerged. Based on the data collected by the information terminal, connecting the Internet, the Internet of Things (IoT), and the mobile communication network, “intelligent aging care” uses new information technology, such as big data and biometrics, to establish a service and interaction platform. Intelligent aging care integrates public and social service resources to meet the needs of the elderly in life care, security, medical attention, spiritual consolation, and other aspects so as to provide a new aging service solution for old people.

In order to support the popularization of intelligent aging care and promote the development of high-quality aging services, the State Council of China issued the “Guidance on actively promoting Internet action” in 2015 and set the target task of boosting the development of the intelligent aging health care industry. In 2017, government departments of aging services jointly published the “Action plan for the development of the intelligent aging health care industry (2017–2020),” which calls for accelerating the research and development of key technologies and products for intelligent, healthy elderly care, advocating the priority use of these products in nursing homes and medical institutions, and encouraging the government to subsidize families and individuals to purchase these products. In 2019, the general office of the State Council of China issued the “Opinions on promoting the development of aging services,” providing suggestions for promoting the integration of home, community, and institutional aging care. On this basis, China implements the intelligent aging service, aiming to continuously push the development of the intelligent aging care industry and explore the application expansion of high-tech technology in the field of aging care [2,3].

**Figure 1:**
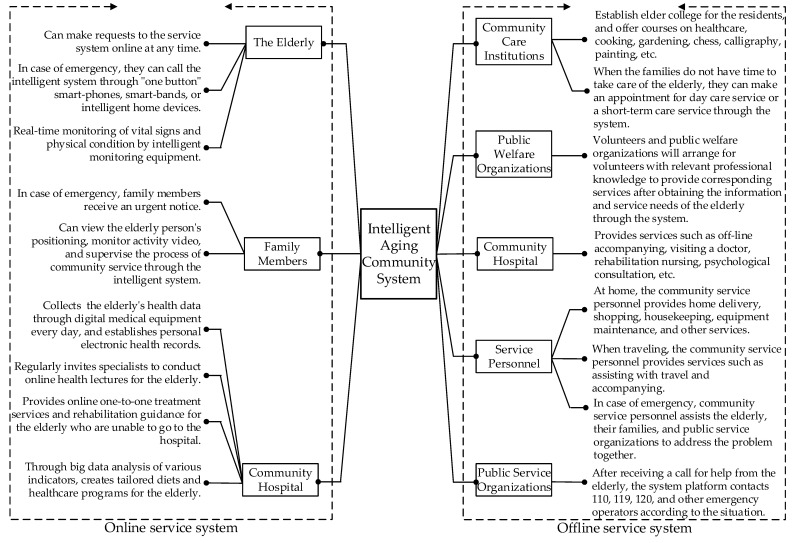
Service system of intelligent aging community [5].

Traditional old-age care in China can be divided into three types: home-based care, community-based care, and institutional care. As a new elderly care model integrating home-based, community-based, and institutional care, the “intelligent aging community” is an important direction for China, promoting the development of aging services, and has introduced the concept of “intelligent” service management. Relying on the intelligent system as the service platform, the intelligent aging community connects the residents, service personnel, hospitals, and social public service institutions, forming a highly efficient, high-quality, multi-level elderly service management system [4]. The operation mode of intelligent aging community service is shown in Figure 1. It can be seen that it focuses on bringing a more comfortable service experience to the elderly, providing a wide range of intelligent services through online and offline service systems to meet the physiological and psychological needs of old people.

Although the technical level of the intelligent aging community is constantly improving, and the operation mode has been continuously perfected during long-term practice, the difference between its goal and its current state has caused a lack of public trust in the intelligent aging community. Public trust determines the speed of industrial development. In order to achieve the large-scale and healthy development of the intelligent aging care industry, it is necessary to improve the public trust level first. Therefore, clarifying the mechanism of establishing public trust in the intelligent aging community is a basic theoretical problem to be solved in the development of the intelligent aging care industry.

Since the concept of intelligent aging care has been announced, it has been in the focus of multiple different industries. By analyzing the policies issued by the central government in recent years, the future development of China’s intelligent aging care industry can be anticipated [6]. At the same time, an evaluation of the emerging gerontechnology industry can highlight regional differences in the development of China’s intelligent aging care industry, allowing local governments to formulate different development policies [7]. From a technical point of view, the design of an intelligent aging care scheme based on the IoT can alleviate the challenges caused by the aging population, such as in-home health care devices [8], smart housing [9], intelligent elderly buildings [10] and so on. In addition, intelligent technologies, such as ambulatory monitoring [11], fall prevention [12], and ambient assisted living [13] also bring great benefits to the life of the elderly. Many scholars have developed and designed intelligent integrated systems in order to provide user-friendly solutions for elderly users [14,15]. From the perspective of service, some large Chinese cities have started to provide intelligent elderly care services and accumulated relevant practical experience since 2015 [16]. However, the service system is not perfect, the supply of services is insufficient, and there is a gap between supply and demand, and demand exceeds supply [17]. In the future, the development of intelligent services should be oriented toward the needs of the elderly, and basic needs, such as life, spiritual needs, and healthcare, should be well met [18]. Moreover, there are numerous problems in other fields related to intelligent aging care that need to be paid attention to, such as service quality management and operations management [19].

In addition, research in Australia has shown that Internet access is also becoming increasingly necessary for the elderly to access information about the government, health, banking, and community services [20]. Moreover, intelligent assistive technologies (IATs) have also been found to be of great help to the Canadian elderly in healthcare, daily life, travel, and communication and is expected to be widely used in the future [21]. Similarly, information and communication technologies (ICTs) innovation for old people will also help the Japanese cope with the challenges of its rapidly aging population [22]. It is evident that intelligent aging care is an effective means to alleviate the aging problem in many countries.

The purpose of the prior research was to improve the capacity limit of intelligent aging services through efficient management and technical means. However, in terms of addressing the gap between the service currently provided and the goal, as well as the impact of customers’ dissatisfaction on public trust, the existing literature has rarely paid attention to the importance of public trust in the provision of intelligent elderly care. For the intelligent aging community, public trust means a positive public attitude towards these community services. It is also an important manifestation of the sound development of the intelligent aging care industry and its social benefits. By studying the formation mechanism of public trust regarding intelligent aging, this paper clarifies the influencing factors and relationships and analyzes the formation processes of public trust so as to clarify the path to service improvement and promote the healthy development of the intelligent aging care industry.

The ACSI model is usually used to evaluate customer satisfaction [23], for example, with online shopping [24] or with public transportation services [25]. This research can be used to calculate the public satisfaction with and trust of community services [26] and can also be used to build a community service system for the elderly from the perspective of customer satisfaction [27]. Most of the existing studies based on the ACSI model have paid greater attention to the evaluation of satisfaction. However, public trust is also an important variable in the ACSI model and will have a positive impact on intelligent elderly care services. Therefore, this article takes public trust as its research focus, analyzing the factors that affect the elderly’s trust in the intelligent care community. Based on the classic ACSI model, this paper establishes a model of the public trust formation mechanism and explores the formation process of public trust in the intelligent elderly care community. By putting forward a hypothesis on the influencing factors on public trust, a structural equation model is constructed, and an empirical test is carried out. The conclusion provides theoretical support for the existing intelligent aging community to improve the residents’ service experience, improve user satisfaction, and increase public trust.

## 2. Materials and Methods

### 2.1. Research Hypothesis

#### 2.1.1. Influencing Factors of Public Trust in the Intelligent Aging Community

Based on the ACSI model, this paper analyzes the influencing factors of public trust in the intelligent aging community from the five dimensions of Perceived ease of use, Perceived quality, Perceived cost economical, Perceived risk, and Public satisfaction.

Perceived ease of use refers to the public’s evaluation of the simplicity of technology when learning or using it. With the advent of the era of science and technology, smartphones and intelligent homes and devices are an integral part of the intelligent senior living community. The difficulty of equipment operation is likely the greatest barrier for the elderly to experiencing the convenience of intelligent service. In the past, the transmission of information has often relied on traditional means, while in the modern, intelligent elderly care community, information transmission mostly depends on intelligent equipment. Due to the individual differences in age and psychological cognition of the elderly, their adaptability to intelligent technology is generally lower than that of young people, and their ability to accept new things is also weaker [28]. Therefore, this paper uses perceived ease of use as an important indicator for measuring the public trust in the intelligent aging community.

After receiving the service, the consumer will have two kinds of perception: the perception of quality and the perception of price, which will be extended to the community service of intelligent elderly care, namely, perceived quality and perceived cost economical [26]. Perceived quality refers to the public’s perception of all aspects of service quality after receiving community services, which is one of the most influential indicators of public trust. Perceived cost economical refers to the public’s evaluation of the quality and the cost of receiving the service. It emphasizes the user’s subjective cognition of the price. Due to the particularity of the consumption behaviors of the elderly, they pay greater attention to the economic cost than young people do. Therefore, this study introduces the concept of perceived cost economical as one of the main factors affecting the public trust in the intelligent aging community.

Perceived risk refers to the adverse consequences that may occur when the public fails to achieve the expected goal after receiving the service. In the traditional elderly community, because of the limited services, the public’s perception of risk is not obvious. However, in the intelligent aging community, due to the integration of smart devices and rich service content, some of the elderly people will inevitably have a skeptical attitude towards it, so the perceived risk should be paid more attention to. At present, the elderly think that the risks involved in services can be roughly divided into three categories. Firstly, personal and financial risks, that is, the threat to their personal safety and that of their property in the service process. Secondly, the functional risk, which means the service function deviates from the comprehension of the elderly or the system is unable to provide the appropriate service. Thirdly, the privacy risk, referring to the risk of personal data being disclosed through the use of smart devices or systems [28].

When accepting intelligent aging care service, if the elderly think that smart devices or systems are effective and easy to use, they will have a better experience of service quality, which has an important impact on perceived quality. On the contrary, if the elderly perceive there to be a higher risk brought about by intelligent service, they will have a more negative attitude toward the service quality. The elderly’s perception of price is based on their perception of quality: the higher the perceived service quality, the more appropriate they will think the cost is. Therefore, this paper makes the following assumptions about the relationships among perceived ease of use, perceived quality, perceived cost economical, and perceived risk:

**Hypothesis** **1.**  Perceived ease of use has a significant positive impact on perceived quality.

**Hypothesis** **2.**  Perceived quality has a significant positive impact on perceived cost economical.

**Hypothesis** **3.**  Perceived risk has a significant negative impact on perceived quality.

Public satisfaction refers to the overall evaluation by the elderly after receiving the intelligent care service. This perception is generated by comparing the products or services provided by the intelligent aging community with the expectations before use. As an important variable in the model, public satisfaction is directly related to all other indicators. For example, perceived ease of use is one of the vital influencing factors; the greater the ease of use of the intelligent equipment, the higher the public satisfaction with the intelligent aging community will be. Similarly, the higher the public’s evaluation of the overall perceived quality and economic viability of the service, the more satisfied users will be with the community service [29,30]. In contrast, if the public thinks that the risk of the community service is high, their overall satisfaction will be reduced. Therefore, the paper tests the following hypothesis about the relationships between perceived ease of use, perceived quality, perceived cost economical, perceived risk, and public satisfaction:

**Hypothesis** **4.**  Perceived ease of use has a significant positive impact on public satisfaction.

**Hypothesis** **5.**  Perceived quality has a significant positive impact on public satisfaction.

**Hypothesis** **6.**  Perceived cost economical has a significant positive impact on public satisfaction.

**Hypothesis** **7.**  Perceived risk has a significant negative impact on public satisfaction.

#### 2.1.2. Formation of Public Trust in the Intelligent Aging Community

Public trust in the intelligent aging community refers to the degree of trust and loyalty of the public to the intelligent care community after receiving the service, which is a reflection of community belonging. Sociologists generally believe that a sense of community belonging is a vital factor for the existence and development of any community [31]. The sense of community belonging refers to the psychological state in which community residents see themselves as belonging to a group of people in a certain region. This kind of psychology includes not only the recognition of their own community identity but also the individual emotion, including their devotion, love, and attachment to the community. Generally, intellectualization will not weaken people’s sense of community belonging. On the contrary, the conditions of material life, cultural life, environment, resources, and other factors that constitute the quality of community have a profound impact on people’s community awareness and sense of belonging. If the conditions of the community cannot meet the residents’ hopes and requirements, it will affect whether the residents are willing to stay in the community. Thus, it can be seen that the sense of community belonging is closely related to public satisfaction. Previous studies have shown that the greater the residents’ satisfaction with the elderly community is, the stronger their trust is in the community [27,32]. Based on this, the paper assumes that:

**Hypothesis** **8.**  Public satisfaction has a significant positive impact on the public trust in the intelligent aging community.

**Figure 2:**
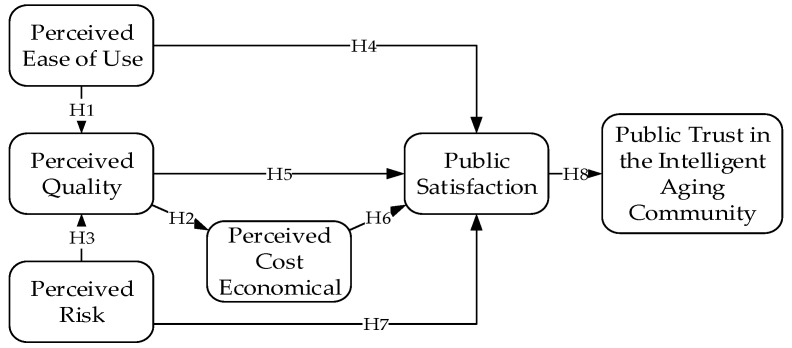
Theoretical model.

According to this hypothesis, the public trust model of the intelligent aging community is proposed, as shown in Figure 2.

### 2.2. Empirical Research Methods

#### 2.2.1. Sample Selection and Data Collection

The empirical data were obtained through a questionnaire survey. According to the “List of the first batch of intelligent and healthy elderly care model communities,” released by the State Council of China in 2017, the representative community in the Changning District of Shanghai was selected as the survey site. The model community has been in operation for more than three years. The intelligent aging service was launched earlier, the service system works relatively well, and the service network layout is reasonable. The interviewees were all elderly people living in these areas and receiving intelligent community care services. They have, thus, formed a better informed subjective evaluation of the service level. A total of 400 questionnaires were distributed in Hongqiao, Tianshan, Beixinjing, and other streets in the model district, and 337 were recovered, with a recovery rate of 84.25%. After further sorting out surveys with missing and substandard responses, 306 questionnaires remained, with a qualification rate of 90.8%.

#### 2.2.2. Research Variables

The paper draws on the existing scale from the relevant research literature and adjusts it according to the situation of this study. In this paper, a 7-point Likert scale was used to measure the relevant variables; 1–7 reflects the degree of agreement with the content described regarding the practical application of the intelligent elderly community: 1 represents a very low degree, and 7 represents a very high degree of agreement. The scale is divided into six parts, with 35 items in total. Unqualified items were deleted through confirmatory factor analysis (CFA), and the contents of the scale are shown in Table 1.

#### 2.2.3. Reliability and Validity Test

The reliability and validity of each item should be evaluated before theoretical hypothesis verification [34]. In this paper, a confirmatory factor analysis (CFA) was carried out for each factor in the model and adjusted according to the factor load and correction index. The CFA results after deleting the items that did not meet the standard are shown in Table 2. In the latent variables of the model, perceived ease of use, perceived cost economical, perceived risk, public satisfaction, and public trust in the intelligent aging community are all first-order while perceived quality is second-order. Therefore, in order to ensure the necessity and rationality of the existence of the second-order factor of perceived quality, the CFA of the multi-factor complete correlation model and the second-order factor model were compared, and the fitness of the two models was calculated [35]. The output results showed that there was no significant difference in the fitness indexes, such as $\chi^{2}/df$, GFI, AGFI, and RMSEA. The target coefficient after comparing the two $\chi^{2}$ was 0.827, which was close to 1, indicating that the second-order factor model could replace the multi-factor complete correlation model. Therefore, the second-order factor of perceived quality met the requirements of the theoretical model [36]. In the model, the combination reliability (CR) of latent variables ranged from 0.768 to 0.897, which were higher than the reliability level of 0.7. The factor load was between 0.637 and 0.914, which was higher than the significance level of 0.6. In addition, the average variation extraction amount (AVE) was 0.526–0.745, greater than the standard level of 0.5. The correlation coefficient matrix is shown in Table 3. The maximum square value of the matrix is 0.511 (0.715 × 0.715), which is less than the minimum AVE value of 0.526. Therefore, this model has good discriminant validity. In conclusion, the scale of this study has passed the reliability and validity tests and can be used to test the model.

## 3. Results

### 3.1. Overall Test of Structural Equation Model

**Figure 3:**
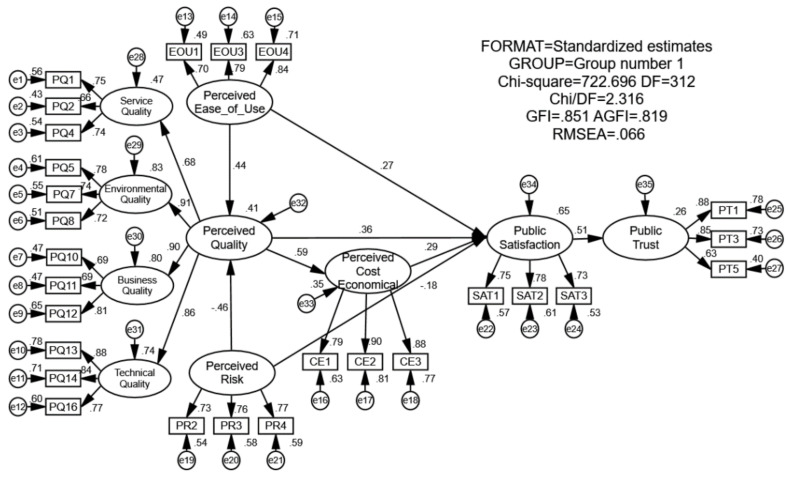
Overall inspection structural equation model.

For this paper, the Amos 21.0 software (Amos 21.0 is a part of IBM SPSS Statistics, which is created by the International Business Machines Corporation (IBM) set in New York, USA) was used to construct the public trust model of the intelligent aging community. The operation results of the structural equation model are shown in Figure 3. The ratio of $\chi^{2}$ to the degree of freedom (CMIN/DF), goodness of fit index (GFI), modified goodness of fit index (AGFI), incremental fitness index (IFI), comparative fitness index (CFI), and root mean square error of approximation (RMSEA) were selected to measure the model fitness. The relevant indicators are shown in Table 4. It can be seen that all indicators meet the standard of the recommended value, so the model match is good.

### 3.2. Direct Effect Path Test

The direct path coefficient test results of the structural equation model are shown in Table 5. The eight direct paths among the six latent variables are significant at the level of *p* < 0.01. In addition, from the structural equation model diagram, it can be seen that there are not only significant direct effects among the variables affecting public trust in the intelligent aging community but also some indirect effects. Therefore, the indirect effects of the model were also tested.

### 3.3. Indirect Effect Path Test

This paper adopts the bootstrapping test method to test the mediating effect of the paths among influencing factors of public trust. The test results are shown in Table 6. The estimated Z-value of the indirect effect between perceived quality and public satisfaction is 2.804, which is greater than the standard value of 1.96 [37]. Meanwhile, at the 95% confidence level, the indirect effect confidence intervals of the bias-corrected percentile method and the percentile method do not contain 0, indicating that there is a significant indirect effect between perceived quality and public satisfaction. Combined with the results of the direct effect path test, there exists a partial mediating effect. Similarly, perceived ease of use and perceived risk have significant partial mediating effects with the path of public satisfaction.

In the influence path of perceived ease of use and perceived risk on public satisfaction, two different mediating effects may exist: perceived quality as the intermediary variable, or perceived quality and perceived cost economical as dual mediating variables. When there are more than two mediating variables, each one may affect the path between the independent and the dependent variables or jointly influence it. Then a specific indirect effect will be produced [38]. Since the bootstrapping test cannot calculate specific indirect effects, this paper adopts writing code to test specific indirect effects through Amos 21.0 software. The test results are shown in Table 7. In the influence path of perceived ease of use and perceived risk on public satisfaction, perceived quality as a single intermediary or perceived quality, and perceived cost economical as dual mediating variables, their indirect effects on the path are both significant.

## 4. Discussion

Based on the empirical study of the formation mechanism of public trust in the intelligent aging care community, this paper clarifies the influencing factors of public trust and the relationship between the influencing factors and sorts out the public trust formation process. The research results show that public satisfaction, perceived quality, perceived cost economical, perceived ease of use, and perceived risk have an important impact on public trust. Among them, perceived risk has a negative impact on public trust, while the other four variables have a positive impact on it. This reflects that by improving service quality, cost economical, ease of use, or reducing service risks, the satisfaction and public trust of the intelligent aged care community can be improved to varying degrees. In addition, through the model test and indirect effect report, it was found that there are obvious indirect effects in the model. Public satisfaction directly affects public trust, while other variables indirectly affect public trust through public satisfaction. Indirect effects show the interaction of various variables in the model, reflecting a variety of ways to increase public trust in the intelligent elderly care services. The research conclusions clarify the path to service improvement for government welfare departments, aging care enterprises, communities, and other institutions, to improve the elderly’s quality of life so as to obtain more trust of the elderly in intelligent services. Moreover, the promotion of public trust will also help the widespread promotion of intelligent aging care services in the future so that more elderly people can experience intelligent care services. This will not only help the intelligent development of the aging care industry but also the government in reducing the burden of pensions.

However, in the research on public satisfaction with community services, results show that among the variables that affect public satisfaction, the impact of the perceived value is stronger, and that of perceived quality is weaker [26]. This is contrary to the results of this study. Since this paper takes the intelligent elderly care community as the research object, it is quite different from the service consumers, methods and contents of an ordinary community [27]. Therefore, the public’s emphasis on community evaluation is different, resulting in different research results. Moreover, unlike the traditional aging care community satisfaction model, this research understands public trust as goal-oriented and adds two variables, namely, perceived ease of use and perceived risk, which are unique to the intelligent elderly care community. The research theme is more in line with the future development direction of China’s elderly care market.

The geographic area this article focuses on is the Changning District, Shanghai. Although the survey scope is relatively small, Shanghai is one of the earliest cities in China to offer intelligent elderly care services, and its service has been relatively standardized. An investigation in this area can better reflect the existing problems and needs for improvement in intelligent elderly care services in China at the present stage. According to the research results, the four factors of perceived quality, perceived cost economical, perceived ease of use, and perceived risk affect the formation of public trust, from strongest to weakest. As Shanghai is one of the most economically developed cities in China, with a relatively high per capita income and per capita education level, the elderly in this region pay the most attention to the quality of intelligent elderly care services, followed by the cost of purchasing such intelligent services. Moreover, Shanghai has a high degree of technology use, many elderly people’s daily lives are inseparable from smart devices, such as mobile payments, online shopping, social networking, and so on. It can be seen that the surveyed residents have a higher acceptance of smart devices and are gradually familiar with how to operate them, so they pay less attention to ease of use. In addition, intelligent aging care services in the Shanghai area have been in operation for many years, and their regulatory and technical systems are relatively more complete than those of other cities. In the service process, there are few personal or property losses, which leads the elderly to believe that intelligent elderly care services bear little risk for them. Therefore, the elderly residents of this area pay less attention to perceived risk than to other factors.

There are also many cities in China that have just begun to provide intelligent elderly care services with less service content and weak supervision, and fewer people receiving these services. If surveys were conducted in these cities, the elderly may not have a deep understanding of intelligent services and may not be able to produce objective and accurate evaluations. With the accumulation of development experience, the progress of technology, and the improvement of supervision in these cities, multiple cities should be selected for investigation in the future research in order to expand the scope of the research and to verify the universality of the model. Future research should also be based on the existing or improved models, and through cross-regional comparative studies, they may reflect that the influencing factors and influence levels of public trust in intelligent aging communities vary in different regions. For example, elderly people in more economically developed areas may pay more attention to service quality rather than economic costs, while elderly in underdeveloped areas may pay more attention to cost-effectiveness than to service quality. In addition, the elderly in developed areas may pay less attention to the ease of use and risks brought about by smart devices, while the old people in traditional areas may pay more attention to ease of use and risks. Therefore, according to the research results of different regions, the government, service institutions, and communities can identify solutions according to local conditions, thereby more effectively improving the public trust in the intelligent aging community. Moreover, according to the theory and model applied in this research, longitudinal comparisons can be carried out in the future. According to the results of future longitudinal research, the development route can be summarized, and the focus of different development stages of intelligent elderly care services can be analyzed. These conclusions can then provide guidance to cities that are in the development stage or have not yet launched intelligent elderly care services, helping them to make continuous progress, and also have important practical significance for the development of China’s elderly care services.

## 5. Conclusions and Suggestions

Based on the ACSI model, this paper constructs the public trust formation mechanism model of the intelligent aging community, obtains data through a questionnaire survey, and empirically tests the model by using a structural equation model. It arrived at the following conclusions:

(1) Public satisfaction has a direct impact on public trust, and public satisfaction has a completely intermediary role in the relationship between other influencing factors and public trust; that is, to improve public trust, public satisfaction must be enhanced first.

(2) Among the influencing factors of public trust in the intelligent aging community, perceived quality and perceived cost economical play a partial mediating role in the interaction between perceived ease of use, perceived risk, and public satisfaction. That is to say, an increase in perceived ease of use and a decrease in perceived risk cannot only directly promote the improvement of public satisfaction but can also improve it through perceived quality, or the joint effect of perceived cost economical and perceived quality.

(3) In addition to public satisfaction, perceived quality is the most important factor affecting public trust in the intelligent aging community, followed by perceived cost economical, perceived ease of use, and perceived risk. It can be seen that the improvement of community quality and cost economical are the fundamental reasons for forming high levels of public trust. In addition, the enhancement of perceived ease of use and the reduction of perceived risks are also significant ways to increase public trust in the intelligent aging community.

According to the research results of the formation mechanism of public trust in the intelligent aging community, the following suggestions are made to enhance public trust:

(1) At the enterprise level, improving the service quality and increasing the elderly customers’ satisfaction with the intelligent aging community is an important way to enhance public trust. Therefore, the operators of intelligent service should adhere to trust-oriented service delivery, establish public trust through reasonable service costs, attract more elderly customers, form economies of scale, and further reduce costs to form a virtuous circle. In addition, the operators should reasonably publicize the service quality so as to avoid a decline in satisfaction and destruction of public trust caused by the excessive promotion of service quality expectations of elderly customers.

(2) From the industrial point of view, the intelligent aging care industry should pay full attention to other industries that intersect with the aging industry, such as the tourism industry, the health care industry, and the cultural industry. Through the mutual use of resources, the intelligent aging care industry should provide diversified service content for elderly customers. From the demand side, the industry should allocate aging care resources and, based on individual needs and the realization of intelligent precision service, reflect the “intelligent” aging characteristics so as to improve the customers’ perceived quality of intelligent aging care. Therefore, it is necessary to make full use of China’s developed communication network and fully integrate the essential resources of the aging industry while enhancing the elderly customer’s satisfaction regarding perceived quality in order to improve the public trust of the intelligent aging community.

(3) From the government’s perspective, it is still up to the government to make efforts to enhance the credibility of the intelligent aging industry. Therefore, the government should rely on its own influence to lead the establishment of regional “intelligent aging community resource platforms,” inspect the quality of resources and fulfill their regulatory responsibility in order to form the operation mode of the government gathering resources and the market allocating resources in the intelligent aging industry. In addition, the government should adopt the necessary financial means to encourage intelligent aging care enterprises to innovate in the field of intelligent equipment, improve the usability of complex, intelligent products, and raise the public trust in the intelligent aging community by using the mechanism of perceived ease of use increasing public trust.

## Figures and Tables

**Table 1:** Latent variables scale design.

Variable		Item		Ref.
Perceived Quality ^1^	Service Quality	PQ1	Convenience of online services	[29,33]
		PQ2	Quality level of interactive services	
		PQ4	Diversification of information channels	
	Environmental Quality	PQ5	Clean and comfortable environment	
		PQ7	Equipment humanization	
		PQ8	Completeness of medical equipment	
	Business Quality	PQ10	Professional technical level of service personnel	
		PQ11	Communication skill of service personnel	
		PQ12	Information disclosure	
	Technical Quality	PQ13	Online response speed of system	
		PQ14	System routine maintenance level	
		PQ16	Diversity of search functions of system	
Perceived Ease of Use ^2^		EOU1	Ease of reading the service contents and health indicators	[28]
		EOU3	Ease of learning to operate smart devices	
		EOU4	Ease of finding the required service in the system	
Perceived Cost Economical ^3^		CE1	Degree to which service price and service quality match	
		CE2	Degree to which consumption capacity and service price match	
		CE3	Degree to which consumption capacity and smart device prices match	
Perceived Risk ^4^		PR2	Hidden dangers of the service to personal and property safety	
		PR3	Disclosure of user data or health data by the system	
		PR4	Inaccurate health data measured by smart devices	
Public Satisfaction ^5^		SAT1	Overall satisfaction	[26]
		SAT2	Satisfaction compared to expectations	
		SAT3	Satisfaction compared to ideal	
Public Trust in the Intelligent Aging Commuity ^6^		PT1	Level of trust in the realization of services in the community	[26,32]
		PT3	The possibility of reusing intelligent aging community services	
		PT5	The possibility of living in an intelligent aging community in the future	

^1^ PQ: Perceived Quality; ^2^ EOU: Perceived Ease of Use; ^3^ CE: Perceived Cost Economical; ^4^ PR: Perceived Risk; ^5^ SAT: Public Satisfaction; ^6^ PT: Public Trust in the Intelligent Aging Community.

**Table 2:** Confirmative factor analysis statement.

Latent Variable		Faceted Items	Factor Loading		C.R. ^2^	AVE ^3^
			MIN	MAX		
Perceived Quality	Service Quality	PQ-1,2,4	0.652	0.774	0.768	0.526
	Environmental Quality	PQ-5,7,8	0.692	0.839	0.807	0.584
	Business Quality	PQ-10,11,12	0.779	0.904	0.881	0.713
	Technical Quality	PQ-13,14,16	0.737	0.768	0.795	0.563
Perceived Ease of Use		EOU-1,3,4	0.699	0.854	0.823	0.609
Perceived Cost Economical		CE-1,2,3	0.796	0.914	0.897	0.745
Perceived Risk		PR-2,3,4	0.723	0.785	0.798	0.568
Public Satisfaction		SAT-1,2,3	0.738	0.849	0.835	0.628
Public Trust in the Intelligent Aging Community		PT-1,3,5	0.637	0.895	0.842	0.645
Recommended Value ^1^			>0.6	<1	>0.7	>0.5

^1^ The recommended values refer to reference [34]; ^2^ C.R.: Combination Reliability; ^3^ AVE: Average Variation Extraction.

**Table 3:** Latent variable correlation coefficient matrix.

Variable	EOU	PT	PR	SAT	CE	PQ
EOU	1.00					
PT	0.536	1.00				
PR	–0.504	–0.400	1.00			
SAT	0.656	0.473	–0.610	1.00		
CE	0.679	0.465	–0.580	0.691	1.00	
PQ	0.564	0.484	–0.587	0.715	0.579	1.00

**Table 4:** Overall test report of structural equation model.

Fitness Index	*χ*^2^/DF ^2^	GFI ^3^	AGFI ^4^	IFI ^5^	CFFI ^6^	RMSEA ^7^
Report Value	2.316	0.851	0.819	0.913	0.913	0.066
Recommended Value ^1^	<3	>0.8	>0.8	>0.9	>0.9	<0.08

^1^ The recommended values refer to reference [35]; ^2^ DF: Degree of Freedom; ^3^ GFI: Goodness of Fit Index; ^4^ AGFI: Modified Goodness of Fit Index; ^5^ IFI: Incremental Fitness Index; ^6^ CFI: Comparative Fitness Index; ^7^ RMSEA: Root Mean Square Error of Approximation.

**Table 5:** Direct effect path analysis report ^1^.

Direct Effect Path	Standardized Path Coefficient	S.E.	*t*-Value	*p*	Hypothesis	Path
						Significance
Perceived Quality ← Perceived Ease of Use	0.444	0.039	5.999	***	H_1_	Significant
Perceived Cost Economical ← Perceived Quality	0.593	0.142	7.116	***	H_2_	Significant
Perceived Quality ← Perceived Risk	–0.463	0.044	–5.928	***	H_3_	Significant
Public Satisfaction ← Perceived Ease of Use	0.265	0.051	4.158	***	H_4_	Significant
Public Satisfaction ← Perceived Quality	0.363	0.142	3.899	***	H_5_	Significant
Public Satisfaction ← Perceived Cost Economical	0.285	0.059	4.321	***	H_6_	Significant
Public Satisfaction ← Perceived Risk	–0.175	0.057	–2.685	**	H_7_	Significant
Public Trust in the Intelligent Aging Community	0.507	0.101	7.520	***	H_8_	Significant
← Public Satisfaction						

^1^ At the significance level of 0.05, *** represents *p* < 0.001, ** represents *p* < 0.01, * represents *p* < 0.05.

**Table 6:** Indirect effect test report ^1.^

Path	Interaction Type	Point Estimates	Product of Coefficients		Bootstrapping–95% CI			
					Bias-Corrected		Percentile	
			SE	*Z*	Lower	Upper	Lower	Upper
Public Satisfaction ← Perceived Ease of Use	Total effect	0.399	0.065	6.138	0.271	0.531	0.275	0.535
	Indirect effect	0.188	0.059	3.186	0.093	0.319	0.093	0.321
	Direct effect	0.211	0.067	3.149	0.070	0.339	0.069	0.338
Public Satisfaction ← Perceived Quality	Total effect	0.813	0.153	5.314	0.555	1.150	0.561	1.162
	Indirect effect	0.258	0.092	2.804	0.106	0.487	0.074	0.446
	Direct effect	0.554	0.168	3.298	0.260	0.914	0.276	0.939
Public Satisfaction ← Perceived Risk	Total effect	–0.366	0.079	−4.633	–0.546	–0.224	–0.531	–0.217
	Indirect effect	–0.214	0.062	−3.452	–0.372	–0.115	–0.355	–0.111
	Direct effect	–0.152	0.068	−2.235	–0.294	–0.023	–0.286	–0.019

^1^ The sample size is 2000, and the confidence level is 95%.

**Table 7:** Specific indirect effect test report ^1^.

Path	Substructure	Point Estimates	Product of Coefficients		Bootstrapping-95% CI			
					Bias-Corrected		Percentile	
			SE	Z	Lower	Upper	Lower	Upper
SAT←EOU	EOU→PQ→CE→SAT	0.060	0.024	2.500	0.025	0.129	0.017	0.111
	EOU→PQ→SAT	0.128	0.054	2.370	0.047	0.261	0.048	0.263
SAT←PR	PR→PQ→CE→SAT	−0.068	0.025	−2.720	−0.139	−0.031	−0.118	−0.021
	PR→PQ→SAT	−0.146	0.060	−2.433	−0.293	−0.059	−0.286	−0.058

^1^ The sample size is 2000, and the confidence level is 95%.

## Data Availability

The data presented in this study are available on request from the corresponding author. The data are not publicly available due to it relates to the privacy of the survey respondents and is part of an ongoing research.

## References

[1] National Bureau of Statistics of the People’s Republic of China. http://www.stats.gov.cn.

[2] China Government Website Three Departments Issued Notice to Carry Out Intelligent Health Care Pilot Project. http://www.gov.cn/xinwen/2017-08/07/content_5216329.htm.

[3] China Government Website Opinions of the General Office of the State Council on Promoting the Development of Aged Care Services. http://www.gov.cn/zhengce/content/2019-04/16/content_5383270.htm.

[4] Chen L., Lu Q., Qiao J.J. (2016). Research on the Construction of the Wisdom Community Endowment Service System. Popul. J..

[5] Woo J. (2017). Designing Fit for Purpose Health and Social Services for Ageing Populations. Int. J. Environ. Res. Public Health.

[6] Huang J.F., Zhang X.Y. (2020). Smart Elderly Care Industry Policies in China from the Policy Tool-Technology Roadmap Model. Forum Sci. Technol. China.

[7] Huang L.C., Li J., Miao H. (2020). A research on the evaluation of emerging gerontechnology industry and its regional development. Sci. Res. Manag..

[8] Pang Z.B., Zheng L., Tian J., Kao-Walter S., Dubrova E., Chen Q. (2015). Design of a terminal solution for integration of in-home health care devices and services towards the Internet-of-Things. Enterp. Inf. Syst..

[9] Stefanov D.H., Bien Z., Bang W.C. (2004). The smart house for older persons and persons with physical disabilities: Structure, technology arrangements, and perspectives. IEEE Trans. Neural Syst. Rehabil. Eng..

[10] Chen Y.T., Mei H.Y. (2020). Research on Intelligent Pension Building System Based on IOT Technology: Taking Japan as an Example. Archit. J..

[11] Dittmar A., Axisa F., Delhomme G. (2004). New concepts and technologies in home care and ambulatory monitoring. Stud. Health Technol. Inform..

[12] Kosse N.M., Brands K., Bauer J.M. (2013). Sensor technologies aiming at fall prevention in institutionalized old adults: A. synthesis of current knowledge. Int. J. Med Inform..

[13] Blackman S., Matlo C., Bobrovitskiy C., Waldoch A., Fang M.L., Jackson P., Mihailidis A., Nygard L., Astell A., Sixsmith A. (2016). Ambient Assisted Living Technologies for Aging Well: A Scoping Review. J. Intell. Syst..

[14] Wong A.M.K., Chang W.-H., Ke P.-C., Huang C.-K., Tsai T.-H., Chang H.-T., Shieh Q.-Y., Chan H.-L., Chen C.-K., Pei Y.-C. (2012). Technology Acceptance for an Intelligent Comprehensive Interactive Care (ICIC) System for Care of the Elderly: A Survey-Questionnaire Study. PLoS ONE.

[15] Su C.J., Chiang C.Y. (2013). IAServ: An Intelligent Home Care Web Services Platform in a Cloud for Aging-in-Place. Int. J. Environ. Res. Public Health.

[16] Jia Y.J., Wang C. (2020). The Construction of Demand-oriented Smart Home Care Service System. Inner Mongolia Soc. Sci..

[17] Liao C.H. (2019). Solution and Realizing Path of the Overall Problem of Intelligent Aged Care Service. Rev. Econ. Manag..

[18] Sui D.C., Peng Q.C. (2016). “Internet+ Home-based care for Senior Citizens”: Research in Service Model of Intellectual Home-based Care for Senior Citizens. J. Xinjiang Norm. Univ. (Ed. Philos. Soc. Sci.).

[19] Hua Z.S., Liu Z.Y., Meng Q.F., Luo X.G., Huo B.F., Bian Y.W., Li S.J., Yang Y., Jin Q.W. (2016). National strategic needs and key scientific issues of intelligent pension services. Bull. Natl. Nat. Sci. Found. China.

[20] Davern M., Winterton R., Brasher K., Woolcock G. (2020). How Can the Lived Environment Support Healthy Ageing? A Spatial Indicators Framework for the Assessment of Age-Friendly Communities. Int. J. Environ. Res. Public Health.

[21] Boger J., Mihailidis A. (2011). The future of intelligent assistive technologies for cognition: Devices under development to support independent living and aging-with-choice. Neuro. Rehabil..

[22] Obi T., Ishmatova D., Iwasaki N. (2013). Promoting ICT innovations for the ageing population in Japan. Int. J. Med Inform..

[23] Fornell C., Johnson M.D., Anderson E.W., Cha J., Bryant B.E. (1996). The American Customer Satisfaction Index: Nature, Purpose, and Findings. J. Mark..

[24] Zhong W.Z., Xi L.L., Wu R.J. (2014). An Empirical Study of Influential Elements of E-satisfaction Based on ACSI Model. Soft Sci..

[25] Zhang C.Q., Liu Y., Lu W.T., Xiao G.N. (2019). Evaluating passenger satisfaction index based on PLS-SEM model: Evidence from Chinese public transport service. Transp. Res. A Policy Pract..

[26] Zou K., Ma G.S. (2009). Research on Satisfaction Assessment towards Community Service Informationization. China Soft Sci..

[27] Yang X.D., Wu Y.X., Yao J.Y. (2016). Study on Building Endowment Community Service System from the Perspective of Customer Satisfaction. China Soft Sci..

[28] Nie M. (2016). The Study of Influencing Factors that Affect Users’ Adoption Behavior in Smart Home Care Service Sampling of Jinan. Master’s Thesis.

[29] Flores R., Caballer A., Alarcón A. (2019). Evaluation of an Age-Friendly City and Its Effect on Life Satisfaction: A Two-Stage Study. Int. J. Environ. Res. Public Health.

[30] Wu Y.D., Liao J.F., Huang M.J., Wang X.W. (2018). Application of structural equation model to explore the influencing factors of community health service satisfaction. Chin. J. Health Stat..

[31] Shan J.J. (2008). Community attachment and community contentment. Urban Probl..

[32] Zhao D.X., Lu X.J., Liu Z.Q. (2009). An Empirical Study on the Model of China’s Urban Community Residents’ Satisfaction. Soft Sci..

[33] Zhang F., Li D. (2019). Multiple Linear Regression-Structural Equation Modeling Based Development of the Integrated Model of Perceived Neighborhood Environment and Quality of Life of Community-Dwelling Older Adults: A Cross-Sectional Study in Nanjing, China. Int. J. Environ. Res. Public Health.

[34] Dennis L.J., Gillaspy J.A., Rebecca P.S. (2009). Reporting Practices in Confirmatory Factor Analysis: An Overview and Some Recommendations. Psychol. Methods.

[35] Doll W.J., Xia W., Torkzadeh G. (1994). A Confirmatory Factor Analysis of the End-User Computing Satisfaction Instrument. MIS Q..

[36] Lai C.-H., Chiu C.-J., Yang C.-F., Pai D.-C. (2010). The Effects of Corporate Social Responsi-bility on Brand Performance: The Mediating Effect of Industrial Brand Equity and Corporate Reputation. J. Bus. Ethics.

[37] Taylor A.B., Mackinnon D.P., Tein J.Y. (2008). Tests of the ThreePath Mediated Effect. Organ. Res. Methods.

[38] Holbert R.L., Stephenson M.T. (2003). The Importance of Indirect Effects in Media Effects Research: Testing for Mediation in Structural Equation Modeling. J. Broadcast. Electron. Media.

